# Synchronous Bilateral Large Renal Cell Carcinoma Treated With Presurgical Pembrolizumab and Lenvatinib Enabling Unilateral Partial Nephrectomy and Dialysis Avoidance: A Case Report

**DOI:** 10.1002/iju5.70095

**Published:** 2025-09-15

**Authors:** Hiroki Kawabata, Shimpei Yamashita, Yuya Iwahashi, Satoshi Muraoka, Takahito Wakamiya, Yasuo Kohjimoto, Isao Hara

**Affiliations:** ^1^ Department of Urology Wakayama Medical University Wakayama Japan

**Keywords:** bilateral renal cell carcinoma, lenvatinib, partial nephrectomy, pembrolizumab

## Abstract

**Introduction:**

Synchronous bilateral renal cell carcinoma is a rare clinical entity that poses considerable challenges in establishing an optimal treatment strategy.

**Case Presentation:**

A 56‐year‐old woman had synchronous bilateral renal cell carcinoma, with tumors in the right (61 mm) and left (71 mm) kidneys. Systemic therapy with pembrolizumab and lenvatinib was administered for five months, resulting in tumor reduction to 35 mm (right) and 52 mm (left). This facilitated right partial nephrectomy and then left radical nephrectomy to achieve complete tumor resection with preserved renal function and avoidance of dialysis.

**Conclusion:**

Pre‐surgical therapy with pembrolizumab and lenvatinib effectively shrank the initially unresectable renal tumors to enable successful partial nephrectomy. This approach may be viable for patients with large bilateral renal masses.

**Summary**

- In synchronous bilateral renal cell carcinoma, the optimal treatment is unclear.
- In our patient, pre‐surgical pembrolizumab and lenvatinib effectively shrank tumors, enabling partial nephrectomy and subsequent radical nephrectomy while preserving renal function.
- Systemic therapy can sometimes facilitate curative surgery for complex renal tumors.

**Abbreviations**
CT: computed tomography
ICI: immune checkpoint inhibitor
RCC: renal cell carcinoma
TKI: tyrosine kinase inhibitor

## 1 Introduction

Synchronous bilateral renal cell carcinoma (RCC) occurs in approximately 3% of patients, and both tumors should be completely resected [1]. Partial nephrectomy is preferred to preserve renal function, but bilateral RCCs that are large or locally advanced require radical nephrectomy and thus dialysis.

Pre‐surgical systemic therapy to shrink tumors can sometimes facilitate partial resection. Combination regimens incorporating immune checkpoint inhibitors (ICIs) and tyrosine kinase inhibitors (TKIs) have become the standard first‐line therapy for unresectable or metastatic RCC [2, 3, 4]. However, there is insufficient supportive data on their use for patients with bilateral RCC.

Our patient had synchronous bilateral RCC that was initially deemed to be unresectable by partial nephrectomy. However, pre‐surgical therapy with pembrolizumab and lenvatinib enabled successful partial and radical nephrectomy, thus preserving renal function and avoiding dialysis.

## 2 Case Presentation

**FIGURE 1:**
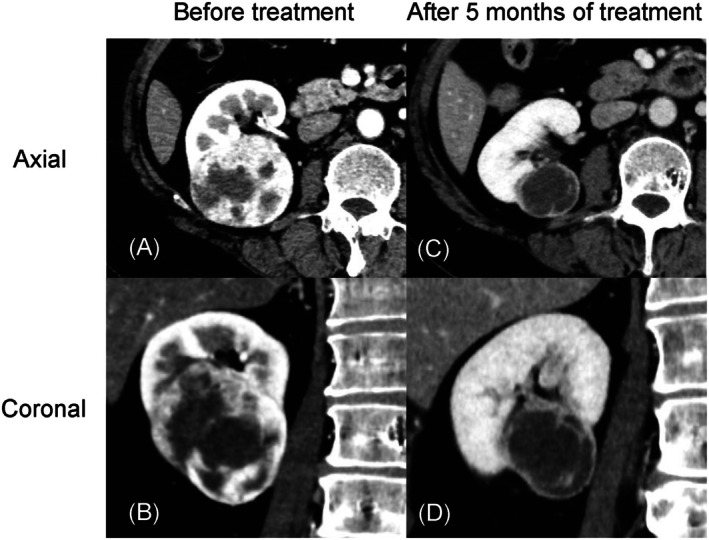
Contrast‐enhanced computed tomography images of the right kidney. (A) Axial view before treatment. (B) Coronal view before treatment. (C) Axial view after 5 months of treatment. (D) Coronal view after 5 months of treatment.

**FIGURE 2:**
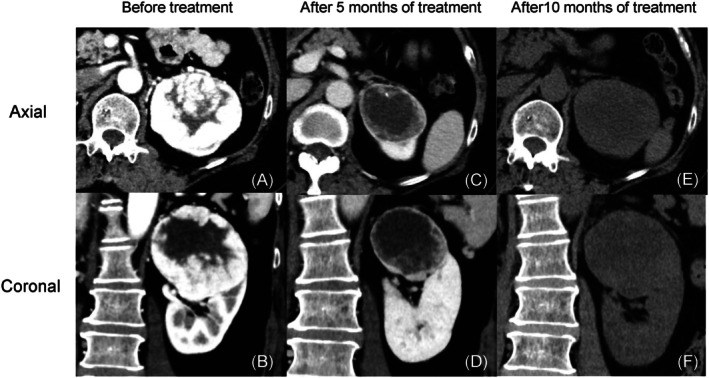
Contrast‐enhanced computed tomography images of the left kidney. (A) Axial view before treatment. (B) Coronal view before treatment. (C) Axial view after 5 months of treatment. (D) Coronal view after 5 months of treatment. (E) Axial view after 10 months of treatment. (F) Coronal view after 10 months of treatment.

A 56‐year‐old woman had gross hematuria and was referred to us for evaluation of synchronous bilateral renal tumors. She had no family history of renal cancer. Contrast‐enhanced computed tomography (CT) revealed a 61‐mm mass in the lower pole of the right kidney (Figure 1A,B) and a 71‐mm mass in the upper pole of the left kidney (Figure 2A,B). Laboratory findings were unremarkable, including a serum creatinine level of 0.66 mg/dL. Imaging suggested bilateral invasion into the renal sinus fat without metastasis, and the clinical stage was cT3aN0M0.

Bilateral radical nephrectomy with dialysis preparation was initially recommended, but the patient strongly preferred to avoid dialysis. We therefore initiated systemic therapy with pembrolizumab (200 mg every 3 weeks) and lenvatinib (20 mg daily), aiming to shrink the tumor enough to allow partial nephrectomy. After 1 month, the patient developed hyperthyroidism, anorexia, and fatigue. The hyperthyroidism, diagnosed as destructive thyroiditis secondary to immune‐related adverse events from pembrolizumab, progressed to Grade 2 hypothyroidism, and it was managed with levothyroxine, allowing continuation of pembrolizumab. Lenvatinib was tapered to 10 mg by the second month due to Grade 2 anorexia and fatigue, and then further reduced to 8 mg by the sixth month due to Grade 2 hand‐foot syndrome.

After 5 months of therapy, contrast‐enhanced CT showed tumor shrinkage of 61 to 35 mm and 71 to 52 mm in the right and left kidneys, respectively (Figures 1C,D and 2C,D). The right kidney tumor had shrunk enough to allow for partial resection, so we decided to discontinue drug therapy and proceed with surgery, rather than attributing this to adverse events. Robotic‐assisted partial nephrectomy via a retroperitoneal approach was performed on the right kidney 9 months after treatment initiation (console time: 244 min, blood loss: 55 mL). Pathology confirmed clear cell RCC, pT1a, Grade 1. Postoperative serum creatinine was 0.83 mg/dL, and renal scintigraphy performed at 1 month postoperatively showed that contributions of the left and right kidneys were 71.9% and 28.1%, respectively. Non‐contrast CT showed regrowth of the left renal tumor to 60 mm (Figure 2E,F), likely due to the discontinuation of systemic therapy 3 weeks prior to surgery.

**FIGURE 3:**
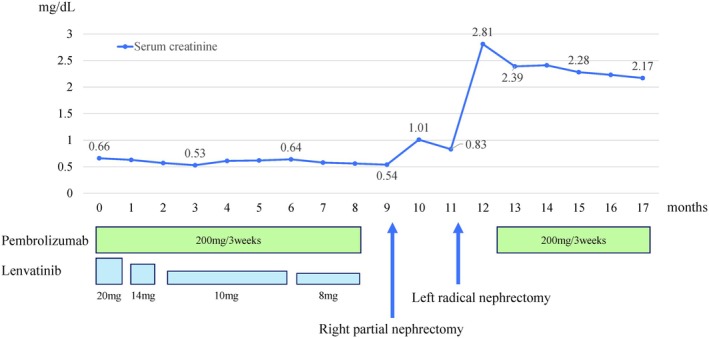
Trends in serum creatinine levels and corresponding drug dosages during the treatment course.

To avoid further adverse events, we did not resume pembrolizumab and lenvatinib because left‐sided surgery was scheduled within 2 months. Radical left‐side nephrectomy was performed 11 months after treatment initiation (console time: 178 min, blood loss: 30 mL). Pathology revealed clear cell RCC, ypT3a, Grade 2. Postoperatively, serum creatinine stabilized at 2.1–2.3 mg/dL (Figure 3), and dialysis was avoided. No adhesions or other indications of drug treatment were observed at the time of either of the surgeries. The patient is currently receiving adjuvant pembrolizumab and remains recurrence‐free 6 months postoperatively.

## 3 Discussion

Presurgical combination therapy enabled partial unilateral nephrectomy in a patient with synchronous bilateral RCC that was initially considered too large and locally advanced for nephron‐sparing surgery. To our knowledge, this is the first reported case of complete surgical resection of synchronous bilateral RCC in which renal function could be preserved following preoperative treatment with pembrolizumab and lenvatinib.

For patients with bilateral RCC without metastasis, complete resection is the standard approach, including partial nephrectomy when feasible. However, when partial resection is not possible, renal replacement therapy may be required, such as dialysis or transplantation. Radical treatments aimed at preserving renal function have been previously described. For example, one reported case involved stereotactic ablative body radiotherapy for a 42‐mm right renal tumor, followed by radical nephrectomy for a 55‐mm left renal tumor [5]. Another reported case involved radical nephrectomy of the right kidney, which contained two tumors (36 and 39 mm), and partial nephrectomy of a 38‐mm tumor in the left kidney performed via ex vivo bench surgery, followed by autologous kidney transplantation [6].

Our patient wanted to avoid hemodialysis, so we initiated pre‐surgical drug therapy, aiming to sufficiently reduce tumor size on one side to permit partial nephrectomy.

Compared with sunitinib, combination therapy with ICIs and TKIs has higher efficacy in patients with metastatic or unresectable clear cell RCC [2, 3, 4]. The combination of pembrolizumab and lenvatinib has been shown in several case reports to produce a favorable reduction in primary renal lesions. For example, in a case of right‐sided RCC with lung metastasis and a 200 mm primary tumor, this combination therapy reduced the tumor to 130 mm, allowing safe deferred cytoreductive surgery [7]. Similarly, pembrolizumab and lenvatinib for a patient with right RCC and level 4 inferior vena cava thrombus reportedly reduced the thrombus to level 2, enabling safe nephrectomy [8]. Furthermore, a patient with right RCC with tumor thrombus in the inferior vena cava received pre‐surgical pembrolizumab and lenvatinib followed by radical nephrectomy and thrombectomy, resulting in a pathological complete response [9].

We therefore elected to use pembrolizumab and lenvatinib for our patient as pre‐surgical therapy, and it resulted in a reduction of the right renal tumor from 61 to 35 mm and downstaging from cT3a to cT1a, which enabled partial nephrectomy. Partial resection of the left renal tumor remained challenging, but its size decreased from 71 to 52 mm. The combination of pembrolizumab and lenvatinib may be effective preoperatively to reduce primary tumor size, and this can perhaps facilitate safer and more feasible radical resection. However, given the variability in half‐lives and adverse effect profiles of TKIs used in combination therapy, further studies are required to establish the optimal regimen for preoperative administration.

## 4 Conclusion

In our patient with large synchronous bilateral RCC, renal function was preserved by pre‐surgical therapy with pembrolizumab and lenvatinib, followed by unilateral partial nephrectomy and contralateral radical nephrectomy. Preoperative administration of pembrolizumab and lenvatinib effectively reduced the size of initially inoperable primary renal tumors, and this facilitated successful surgical intervention with the desired preservation of renal function.

## Ethics Statement

The authors have nothing to report.

## Consent

The authors have nothing to report.

## Conflicts of Interest

Yasuo Kohjimoto is both an Editorial Board member of the International Journal of Urology and a co‐author of this article. To minimize bias, he was excluded from all editorial decision‐making related to the acceptance of this article for publication. The other authors declare no conflicts of interest.
